# Complete genome sequence of *Serratia marcescens* strain CH31 isolated from a *Periplaneta americana* associated with a tertiary hospital setting

**DOI:** 10.1128/mra.00611-25

**Published:** 2025-09-17

**Authors:** Lucia A. Nuncio-García, Mishael Sánchez-Pérez, Angel Andrade, Jesús A. Dávila-Barboza, Faviola Tavares-Carreón

**Affiliations:** 1 Facultad de Ciencias Biológicas, Universidad Autónoma de Nuevo León 27771, Nuevo Leon, Mexico; 2 Centro de Ciencias Genómicas, Universidad Nacional Autónoma de México, Morelos, Mexico; 3 División de Materiales Avanzados, Grupo de Ciencia e Ingeniería Computacionales, Centro Nacional de Supercómputo, Instituto Potosino de Investigación Científica y Tecnológica, San Luis Potosi, Mexico; 4 Departamento de Microbiología, Facultad de Medicina, Universidad Autónoma de Nuevo León 27771, Nuevo Leon, Mexico; The University of Arizona, Tucson, Arizona, USA

**Keywords:** *Serratia marcescens*, insect-vector

## Abstract

Hospital-acquired infections caused by *Serratia marcescens* are steadily increasing worldwide. Here, we report complete genome sequences of *S. marcescens* CH31, isolated from *Periplaneta americana* collected in the periphery of a Mexican tertiary hospital. The 5,627,793 bp genome (58.96% guanine-cytosine) consists of a chromosome and a plasmid.

## ANNOUNCEMENT

*Serratia marcescens*, a versatile opportunistic pathogen in the *Yersiniaceae* family, infects humans, animals, plants, and insects and has caused notable outbreaks in intensive care units (1–4). Recent core-gene-based phylogenetic analyses have revealed significant genomic divergence among *S. marcescens* clusters, suggesting that genetic differentiation is driven by evolutionary adaptation to specific environments or by the emergence of distinct genetic lineages (5–8). While the species is widespread, most sequenced genomes originate from clinical isolates, whereas genetic information from soil, water, or insect-derived strains is scarce. Investigating these less-explored reservoirs is crucial for understanding pathogen evolution, transmission dynamics, and public health risks.

The *S. marcescens* CH31 strain was isolated from the midgut of a hospital-associated *Periplaneta americana* in August 2024. DNase agar supplemented with cephalothin (100 µg/µL) was used for initial isolation and screening. Serial dilutions of the midgut content were plated on DNase agar and incubated at 30°C. One uniform pink colony was then confirmed as *S. marcescens* by PCR using species-specific oligonucleotides for *S. marcescens*: SMA1 (5′ GAACYTGCGCATGATTTATGCG and 3′ CGGWGACGACCTGCAGCTG) and SMA2 (5′ ATGRCCGGYAAGGCCATCGAT and 3′ TTCAGGGCGACCGCGTCG), as previously reported by reference 9. The species identity was confirmed by matrix-assisted laser desorption/ionization time-of-flight VITEK Mass Spectrometry (Biomerieux).

Cells of *S. marcescens* CH31 were grown aerobically at 30°C in Luria-Bertani medium. Approximately 2.0 × 10^9^ cells were collected, and the genomic DNA was purified using a Bacterial DNA Preparation-Column Kit (Jena Bioscience), following the manufacturer’s protocol. Libraries were prepared with the Oxford Nanopore Technologies Rapid Barcoding Kit 24 (v.14) (SQK-RBK114.24). Long-read sequencing was performed using the MinION Mk1B (Software MinKNOW) with a FLO-MIN114 flow cell (Oxford Nanopore Technologies). Raw sequencing data were demultiplexed and basecalled using a Guppy (v.6.4.8) high-accuracy model (10). A total of 94,550 reads were obtained with an average length of 6,000–8,000 bp. Raw reads were processed with Filtlong (v.0.2.1) for quality control and filtering (https://github.com/rrwick/Filtlong). Genome assembly was performed with Trycycler (v.0.5.5) and the assembled genome quality was assessed using QUAST (v.5.2.0). The completeness of the genome was assessed using CheckM2 (v.1.2.2) (11), yielding a value of 100% and 1.27% of contamination. All bioinformatic analyses were performed using default parameters. The genome of *S. marcescens* CH31 comprises 5,627,793 bp with a guanine-cytosine content of 58.96%, achieving a coverage depth of 109× and distributed in two contigs: contig 1 (5,483,021 bp circular chromosome) and contig 2 (144,772 bp linear plasmid). Gene annotation was performed using Prokaryotic Genome Annotation Pipeline (v.6.10), which revealed 5,416 genes (total), 5,203 coding genes, 22 rRNAs, 89 tRNAs, 13 ncRNAs, and 90 total pseudogenes.

**Fig 1:**
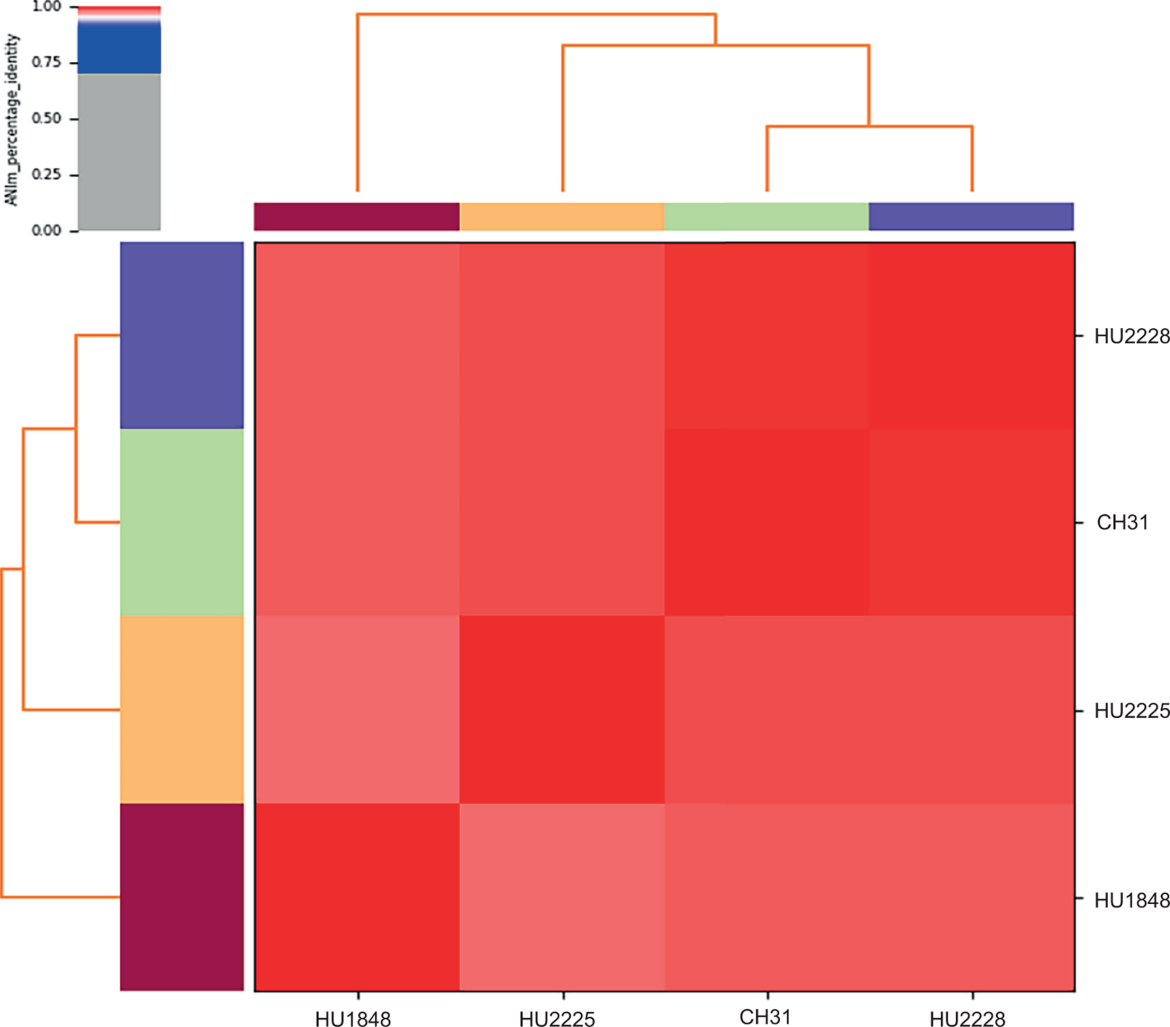
Heatmap of average nucleotide identity (ANI) values from pairwise genomic comparisons. The heatmap, accompanied by a dendrogram, illustrates the phylogenetic relationships based on percentage identity, ranging from high (red) to low (gray), among the complete genomes of *Serratia marcescens* CH31 and the environmental strains HU1848, HU2225, and HU2228. The dendrogram reflects the degree of genomic similarity, with ANI values above 99% indicating that the genomes likely belong to the same species. Accession numbers are provided in the main text.

Average nucleotide identity (ANI)-based phylogeny, generated using the DFAST_QC pipeline (v.0.5.7) with default parameters (12), revealed a close relationship between the 5.4 Mbp CH31 genome and three pigmented *S. marcescens* clinical strains (HU1848, HU2225, and HU2228), previously isolated from patients at a hospital in the same metropolitan area (8) (ANI: 98%, 98%, and 99%, respectively) (Fig. 1). This finding highlights the genetic relatedness between the patient-derived *S. marcescens* isolates and the urban-environmental strain CH31.

## Data Availability

This Whole Genome Shotgun project has been deposited at DDBJ/ENA/GenBank under accession numbers CP192733 (chromosome) and CP192734 (plasmid). The raw sequence reads have been deposited in the Sequence Read Archive (SRX29887937) under the BioProject (PRJNA1266259) and BioSample (SAMN48671768).
